# *In Vitro* and *In Vivo* Evaluation of Zinc-Modified Ca–Si-Based Ceramic Coating for Bone Implants

**DOI:** 10.1371/journal.pone.0057564

**Published:** 2013-03-06

**Authors:** Jiangming Yu, Kai Li, Xuebin Zheng, Dannong He, Xiaojian Ye, Meiyan Wang

**Affiliations:** 1 Department of Orthopaedics, Changzheng Hospital, Second Military Medical University, Shanghai, People’s Republic of China; 2 Key Laboratory of Inorganic Coating Materials, Shanghai Institute of Ceramics, Chinese Academy of Sciences, Shanghai, People’s Republic of China; 3 National Engineering Research Center for Nanotechnology, Shanghai, People’s Republic of China; University of California, Merced, United States of America

## Abstract

The host response to calcium silicate ceramic coatings is not always favorable because of their high dissolution rates, leading to high pH within the surrounding physiological environment. Recently, a zinc-incorporated calcium silicate-based ceramic Ca_2_ZnSi_2_O_7_ coating, developed on a Ti-6Al-4V substrate using plasma-spray technology, was found to exhibit improved chemical stability and biocompatibility. This study aimed to investigate and compare the *in vitro* response of osteoblastic MC3T3-E1 cells cultured on Ca_2_ZnSi_2_O_7_ coating, CaSiO_3_ coating, and uncoated Ti-6Al-4V titanium control at cellular and molecular level. Our results showed Ca_2_ZnSi_2_O_7_ coating enhanced MC3T3-E1 cell attachment, proliferation, and differentiation compared to CaSiO_3_ coating and control. In addition, Ca_2_ZnSi_2_O_7_ coating increased mRNA levels of osteoblast-related genes (alkaline phosphatase, procollagen α1(I), osteocalcin), insulin-like growth factor-I (IGF-I), and transforming growth factor-β1 (TGF-β1). The *in vivo* osteoconductive properties of Ca_2_ZnSi_2_O_7_ coating, compared to CaSiO_3_ coating and control, was investigated using a rabbit femur defect model. Histological and histomorphometrical analysis demonstrated new bone formation in direct contact with the Ca_2_ZnSi_2_O_7_ coating surface in absence of fibrous tissue and higher bone-implant contact rate (BIC) in the Ca_2_ZnSi_2_O_7_ coating group, indicating better biocompatibility and faster osseointegration than CaSiO_3_ coated and control implants. These results indicate Ca_2_ZnSi_2_O_7_ coated implants have applications in bone tissue regeneration, since they are biocompatible and able to osseointegrate with host bone.

## Introduction

In recent years, calcium silicate-based ceramics have become more promising as potential implant biomaterials for bone tissue engineering due to their bioactive and biocompatible properties [1]–[3]. Despite the beneficial influence on bone response, well-known drawbacks associated with this material exist, such as poor mechanical properties, which limit the scope of its clinical application [4]–[6]. However, these limitations can be overcome by surface-modification techniques [7], [8]. The bioactive ceramic coating would confer adequate bioactivity to the surface of the implant, preventing direct contact between the substrate and surrounding bone tissue, thus reducing release of problematic ions from the metallic substrate.

Several coating techniques have been developed and include flame spraying, sputtering, electrophoretic coating, hot isostatic pressing and solution coating [9]. Each approach has its advantages and disadvantages; however, plasma-spraying has shown the most promise as a coating method. In fact, plasma-spraying is reported as the method of choice for coating CaSiO_3_ and Ca_2_SiO_4_ onto metal substrates, both of which have been used clinically to enhance bioactivity and bonding strength with titanium alloy in comparison to HA coating [10]. However, major limitations of calcium silicate coatings include deleterious biological effects due to their high dissolution rate and induction of high pH within the surrounding tissues, which limits further their biomedical application [5].

**Figure 1:**
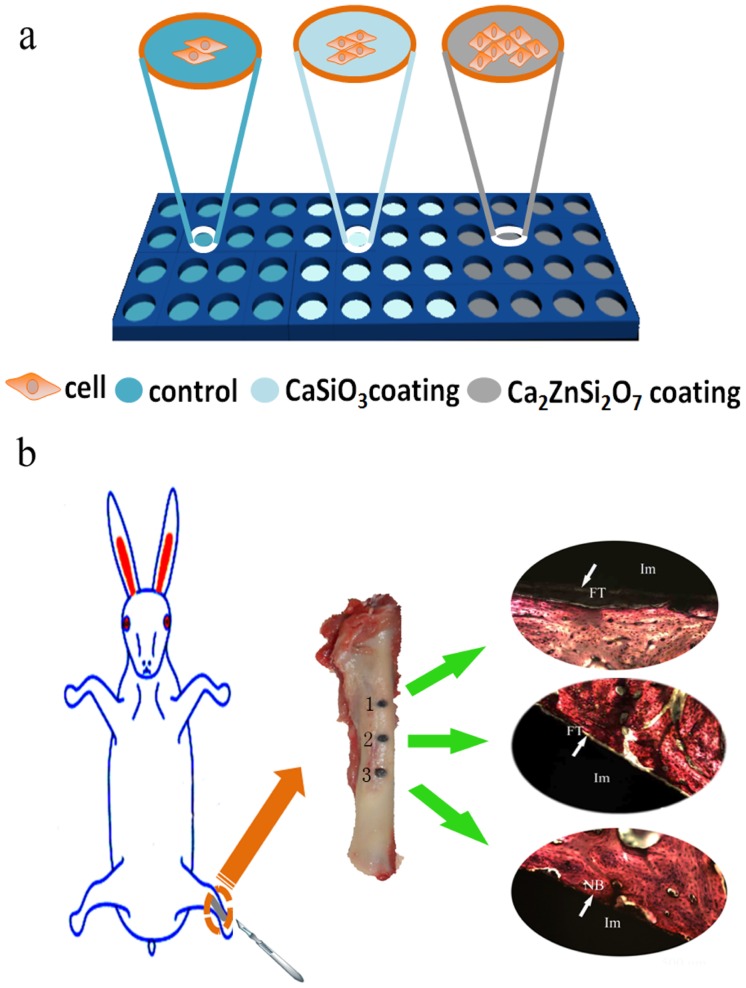
(a) Schematic representation of MC3T3-E1 cell proliferation on uncoated Ti-6Al-4V titanium (control), CaSiO_3_ and Ca_2_ZnSi_2_O_7_ coated substrates. (b) Schematic illustration of *in vivo* analysis. White arrows indicated new bone was formed at the Ca_2_ZnSi_2_O_7_ coating periphery with direct contact to the implant surface in absence of a connective tissue layer in group 3. In contrast, a wide band of fibrous tissue was clearly seen around the surface of the CaSiO_3_ coating and control in group 2 and 1. Group 1, control; Group 2, CaSiO_3_ coating; Group 3, Ca_2_ZnSi_2_O_7_ coating.

Recently, ion-modification of Ca–Si-based ceramics has been developed to improve their chemical stability and biomedical properties and includes divalent (Mg [11], Zn [12], [13] and Sr [14]) and tetravalent (Ti [5] and Zr [15]) modification. Moreover, Ti and Zr-incorporated Ca–Si-based ceramics have been used as stable coatings that improve biomedical properties compared with calcium silicate coatings [16], [17]. However, Zn-modified calcium silicate (Ca_2_ZnSi_2_O_7_) ceramic coatings have not been fully investigated to date. It is well established that zinc, an essential trace element, plays an important role in various physiological processes [18]. Zn has been shown to have a stimulatory effect on bone formation and an inhibitory or biphasic effect on osteoclastic bone resorption [19]. Zinc deficiency results in the arrest of bone growth, bone development, and the overall maintenance of bone health [20], [21]. This indicates Ca_2_ZnSi_2_O_7_ is an interesting biomaterial coating that warrants further investigation. In our previous work, Zn was selected for incorporation into a Ca–Si system to form hardystonite (Ca_2_ZnSi_2_O_7_), which was used as a feedstock coating on the Ti-6Al-4V substrate using plasma-spray technology. Ca_2_ZnSi_2_O_7_ coating exhibited chemically stable (low dissolution) and good bioactivity in comparison with CaSiO_3_ coating [22]. In the present study, we investigate how Ca_2_ZnSi_2_O_7_ coating affects adhesion, morphology, orientation, proliferation and osteoblast differentiation of MC3T3-E1 osteoprogenitor cells (Fig. 1a). The *in vivo* osseointegration potential of Ca_2_ZnSi_2_O_7_ coating was also assessed using a rabbit femur defect implantation model (Fig. 1b).

## Materials and Methods

### Specimen Preparation and Characterization

Ca_2_ZnSi_2_O_7_ powders and coatings were prepared according to methods described previously [22]. Briefly, Ca_2_ZnSi_2_O_7_ powders were synthesized by the sol-gel method using zinc nitrate hexahydrate (Zn(NO_3_)_2_.6H_2_O; Sinopharm Chemical Reagent Co., Ltd (SCRC), China), calcium nitrate tetrahydrate (Ca(NO_3_)_2_.4H_2_O; SCRC, China) and tetraethyl orthosilicate (TEOS, (C_2_H_5_O)_4_Si); SCRC, China). The obtained Ca_2_ZnSi_2_O_7_ powders were sieved through 80 meshes and sprayed onto Ti-6Al-4V substrates with dimensions of 10×10×2 mm and ø10×1 mm, for *in vitro* cell culture and *in vivo* study respectively. The atmosphere plasma spray (APS) system (Sulzer Metco, Switzerland) was used to fabricate all coatings. The thickness of the coating was approximately 170 µm. Before plasma-spraying, substrates were ultrasonically cleaned in acetone and grit-blasted using corundum sand of F60 grade. CaSiO_3_ coatings were prepared using the same conditions as the control. Samples were ultrasonically cleaned and sterilized in acetone, ethanol and distilled water for 10 min.

The phase composition, surface morphology, surface roughness (Ra) and bonding strength of the Ca_2_ZnSi_2_O_7_ coating was characterized in our previous work [22].

### *In vitro* Testing

#### Cell culture

The osteoblast-like cell line, MC3T3-E1, was obtained from the Chinese Academy of Sciences Cell Bank. Cells were cultured in culture plates containing alpha-minimal essential medium (α-MEM, Gibco BRL, Invitrogen, Life Technologies, USA) supplemented with 10% (v/v) fetal bovine serum (FBS) and 1% (v/v) of a 100 U/ml penicillin and 100 µg/ml streptomycin solution (Gibco BRL, respectively). Cells were incubated at 37°C in the presence of 5% CO_2_. On reaching 80% confluence, cells were detached every 2–3 days using 0.25% trypsin/EDTA solution (Gibco BRL).

#### Cell attachment and morphology

MC3T3-E1 cells were seeded onto uncoated Ti-6Al-4V titanium (control), CaSiO_3_ and Ca_2_ZnSi_2_O_7_ coated substrates within individual wells of 24-well culture plates at a density of 1×10^4^ cells/cm^2^. Cells were incubated in α-MEM culture medium supplemented with 10% FBS at 37°C in 5% CO_2_ for 24 h. Samples were rinsed with PBS (pH 7.4) to remove non-adherent cells and fixed in 4% paraformaldehyde for 1 h. Cells were subsequently rinsed with PBS buffer containing 4% (w/v) sucrose and post-fixed in 1% osmium tetroxide in PBS, followed by sequential dehydration in an ethanol series of 30%, 50%, 70%, 90%, 95% and 100% for 5 minutes in each concentration. Specimens were sputter-coated with gold and the morphological characteristics of the attached cells were determined using scanning electron microscope (SEM, S-4800, Hitachi, Japan).

#### Cell proliferation

Cell proliferation was measured using a standard methyl thiazolyl tetrazolium (MTT) assay (Sigma-Aldrich, Saint Louis, MO, n = 6 for each sample). MC3T3-E1 cells were cultured on control, CaSiO_3_ and Ca_2_ZnSi_2_O_7_ coated substrates placed individually in a 24-well culture plate at a density of 1×10^4^ cells/cm^2^ and allowed to grow for 1, 4, 7 and 14 days. At the specified time-points, the substrates were washed in PBS and transferred to another new 24-well plate. MTT stock solution (10% of total volume) was added to 24-well plates containing the coatings and incubated for 4 h at 37°C and 5% CO_2_. Medium was withdrawn and dimethyl sulphoxide (Sigma–Aldrich) was added to each well to dissolve the formazan dye crystals. 100 ul of the reacted reagent from each well was transferred to 96-well plates and the absorbance measured using a microplate reader (SPECTRA MAX PLUS 384 MK3, Thermo Fisher Scientific, Waltham, MA, USA) at a wavelength of 490 nm.

#### Cell differentiation and mineralization

MC3T3-E1 cell osteoblast differentiation and mineralization were determined using cells (1×10^4^ cells/cm^2^) cultured in triplicate (n = 3) on each coating group in differentiation medium comprising α-MEM medium supplemented with 50 µg/ml ascorbic acid (Sigma-Aldrich) and 10 mM β-glycerophosphate (Sigma-Aldrich). Cell differentiation and mineralization were characterized from alkaline phosphatase (ALP) activity and Osteocalcin (OC) secretion. Concentrations of ALP and OC were individually determined using ELISA kits (R&D Systems, Minneapolis, MN, USA) as described below.

#### Quantitative measurement of ALP activity

MC3T3-E1 cells were cultured on control, CaSiO_3_ and Ca_2_ZnSi_2_O_7_ coated substrates in differentiation medium for 1, 4, 7 and 14 days. ALP activity was measured using stable *p*-nitrophenol phosphate substrate. At each time point, culture medium was removed by decantation and cells were washed with PBS and harvested in 1 ml universal ALP buffer (100 mM citric acid, 100 mM KH_2_PO_4_, 100 mM sodium tetraborate.10 H_2_O, 100 mM Tris, 100 mM KCl; pH 11). Cells were sonicated twice for 20 sec and centrifuged at 3000 rpm for 5 min at 4°C. ALP activity in the supernatants was determined following addition of *p*-nitrophenyl phosphate substrate and the reaction was stopped using 100 µl of 0.1 N NaOH. The optical density was measured at 405 nm using a microplate reader (SPECTRA MAX PLUS 384 MK3, Thermo). The ALP activity was calculated from a standard curve after normalization to total protein content, which was measured using the Bradford protein assay kit (Pierce, Rockford, IL, USA). ALP experiments were repeated twice with n = 3 for each substrate.

#### Quantitative measurement of OC secretion

MC3T3-E1 cells were cultured on control, CaSiO_3_ and Ca_2_ZnSi_2_O_7_ coated substrates in differentiation medium for 1, 7, 14 and 21 days. Quantitative levels of OC secreted into the culture medium were determined using an enzyme-linked immunoassay (ELISA) kit (R&D) following the manufacturer’s instructions.

#### Quantitative reverse transcription-PCR (qRT-PCR)

Total RNA was isolated from MC3T3-E1 cells on each coated substrate using Trizol reagent (Fermentas, Maryland, NY, USA). First-strand cDNA synthesis was carried out using Superscript III reverse transcriptase (Invitrogen, Carlsbad, CA, USA) for 60 min at 50°C. Quantitative RT-PCR for MC3T3-E1 cell osteoblast-related genes of alkaline phosphatase (ALP), procollagen α1(I) (Col α1(I)), osteocalcin (OC)and growth factors gene of insulin-like growth factor-I (IGF-I) and transforming growth factor-β1 (TGF-β1), was performed using the ABI PRISMs 7000 Sequence Detection System (Applied Biosystems, Foster City,CA, USA). The amount of target gene transcript in each sample was determined using SYBR® Premix Ex Taq™ II (Takara, Kyoto, Japan) in a final volume of 20 µl containing the same amount of RT product (50 ng cDNA), 10 µl of 2×SYBR® Premix Ex Taq™ II and 0.8 µl of 10 µM forward and reverse primers. Primers for the selected genes are listed in Table 1. Amplification conditions were as follows: Stage 1∶95°C for 30 s; Stage 2∶30 repetitions of 95°C for 5 s, 60°C for 30 s. Each RT-PCR quantification experiment was performed in triplicate for individual samples. Final results were reported as the relative expression normalized with transcript level of the housekeeping gene, Glyceraldehyde 3-phosphate dehydrogenase (GAPDH).

**Table 1:** Primers used for qRT-PCR.

Gene	Sequence(5′–3′)	Melting Temperature(°C)	product length (bp)
GAPDH	F: TCCACTCACGGCAAATTCAACG	60	145
	R: TAGACTCCACGACATACTCAGC		
ALP	F: GCTGATCATTCCCAGGTTTT	60	204
	R: CTGGGCCTGGTAGTTGTTGT		
Col α1(I)	F: TTCTCCTGGTAAAGATGGTGC	60	255
	R: GGACCAGCATCACCTTTAACA		
OC	F: CCTCAGTCCCCAGCCCAGATC	60	220
	R: CAGGGCAGAGAGAGAGGACAG		
IGF-I	F: CACTCATCCACAATGCCTGTCT	60	118
	R: CTGAGCTGGTGGATGCTCTTC		
TGF-β1	F: CCCTATATTTGGAGCCTGGA	60	141
	R: CTTGCGACCCACGTAGTAGA		

Shown are the details of the primers used for qRT-PCR, including melting temperatures, forward (F) and reverse (R) sequences and product length. GAPDH, glyceraldehyde-3-phosphate dehydrogenase; ALP, alkaline phosphatase; Col α1(I), procollagen α1(I); OC, osteocalcin; IGF-I, Insulin-like growth factor-I; TGF-β1, transforming growth factor-β1.

### *In vivo* Analysis

#### Surgical procedure

Total nine White New Zealand rabbits (obtained from Shanghai Jiao Tong University, Laboratory Animal Center, male, 2–2.5 kg body weight) were randomly divided into 3 groups. The use of animals and the experimental protocols was approved by the Institutional Animal Welfare Committee of Shanghai Jiao Tong University. A total of six cylindrical implants of approximately 1 mm in diameter and 1 cm in length were implanted into the femur of each rabbit. Three implants were inserted into the femur of the left hind leg and another three into the femur of the right hind leg. A schedule was prepared to ensure equal placement of the three implant types within each of the six femoral sites and one of each implant types placed into each femur. Rabbits were anesthetized by injecting 3% Nembutal (30 mg/kg) *via* the ear vein and a longitudinal incision was made by scalpel in the rabbit femur under rigorous aseptic conditions. Circular holes, 1 mm diameter by 1.0 cm deep, were drilled using a surgical electronic drill and thoroughly rinsed with physiological saline to remove shards of bone. Implants of Ti–6Al–4V (control), CaSiO_3_-coated Ti–6Al–4V and Ca_2_ZnSi_2_O_7_-coated Ti–6Al–4V were used in this study. Before insertion of implants, blood was removed using sterile cotton balls and the holes were manually filled with implants as tightly as possible. The wound was sutured with nylon thread. Rabbits were sacrificed 1.5 month after implantation.

#### Specimen preparation

After sacrifice, Excised specimens were fixed in 4% paraformaldehyde for 3 days, dehydrated in a series of ethanol solutions (70, 80, 90, 95 and 100%) and embedded in polymethylmethacrylate resin. Undecalcified sections with a thickness of 100 µm were cut using a saw microtome (Leica ST1600) and stained with trinitrophenol-fuchsin prior to histological analysis. A minimum of 6 sections were made from each implant and used for descriptive and morphometric analyses.

#### Histology and histomorphometry

Histological observation and histomorphometrical analysis of the sections were performed under a biologic fluorescence microscope (Olympus BX-60). The microscopic images of the sections were digitized and analyzed using an image-analyzing software (KS400). The bone-implant contact rate (BIC) was used as the index of osseointegration, equal to the surface length contact of the implant with the bone/the overall surface length of implant inserted into the bone.

### Statistical Analysis

For statistical analysis, first Levene’s test was performed to determine the homogeneity of variance for all the data, and then one way analysis of variance followed by Tukey or Tamhane’s T2 post-hoc test for multiple comparison was performed for the comparisons between different groups. SPSS 17.0 program was employed for all statistical analysis and differences were considered significant if *p*<0.05. All data were expressed as a mean ± standard deviation (SD).

## Results

### Cell Attachment and Morphology

**Figure 2:**
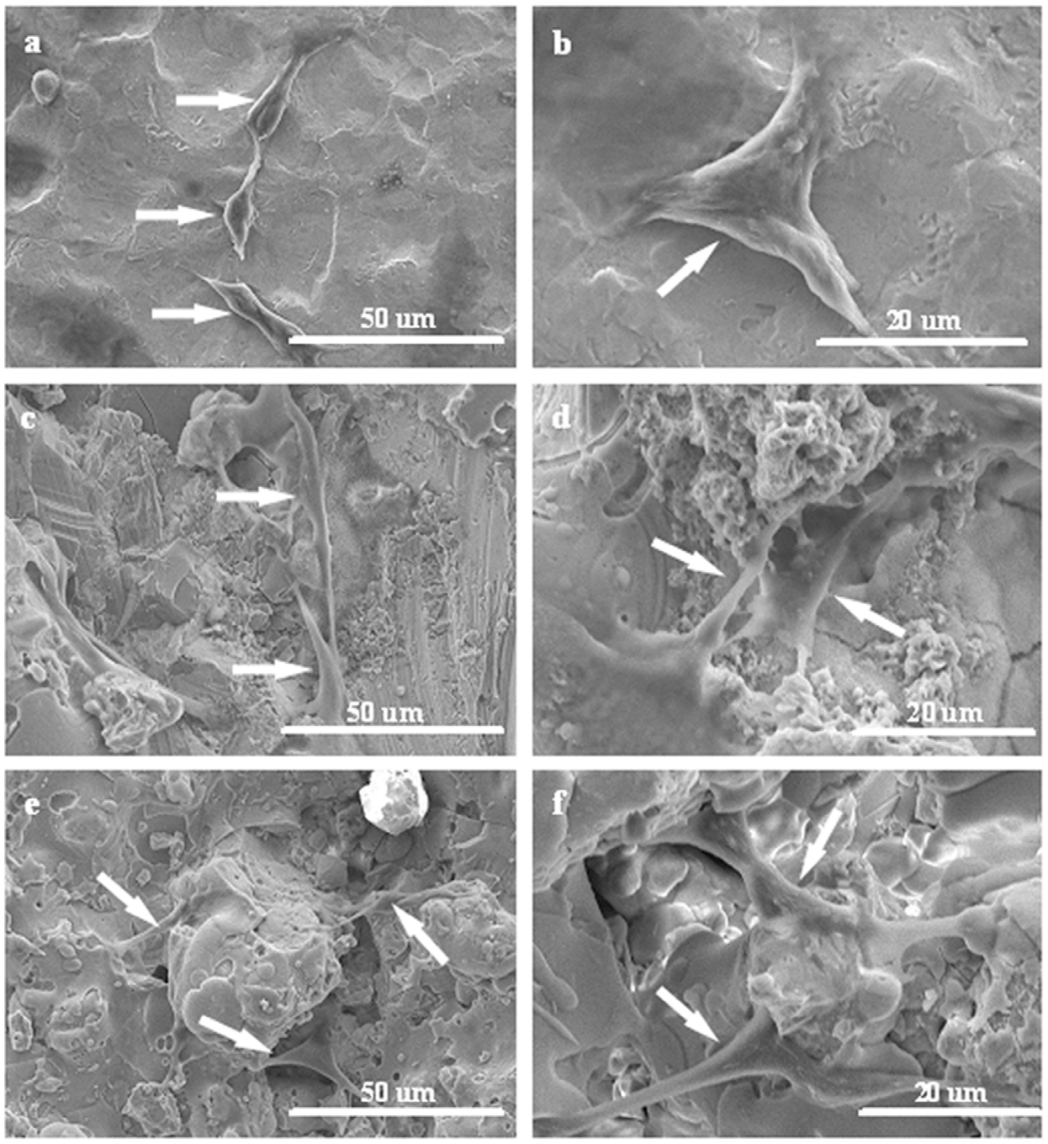
SEM images of MC3T3-E1 cell morphology on the different substrates 24 h after seeding:(a, b) control, (c, d) CaSiO_3_ coating and (e, f) Ca_2_ZnSi_2_O_7_ coating. The arrows indicated cells cultured on the different substrates.

MC3T3-E1 cell attachment and morphology on control, CaSiO_3_ and Ca_2_ZnSi_2_O_7_ coated substrates were examined using SEM (Fig. 2). After 24 h of culture, MC3T3-E1 cells were found to attach to all tested samples surface. The cells displayed a triangle-like morphology on the surface of the titanium control (Fig. 2a, b) but appeared spindle-like on the surface of both the Ca_2_ZnSi_2_O_7_ (Fig. 2e, f) and CaSiO_3_ (Fig. 2c, d) coated substrates. A greater number of cells had cytoplasmic extensions, which were longer in protrusion, on the Ca_2_ZnSi_2_O_7_ coated surface in comparison to CaSiO_3_ and control.

### Cell Proliferation

**Figure 3:**
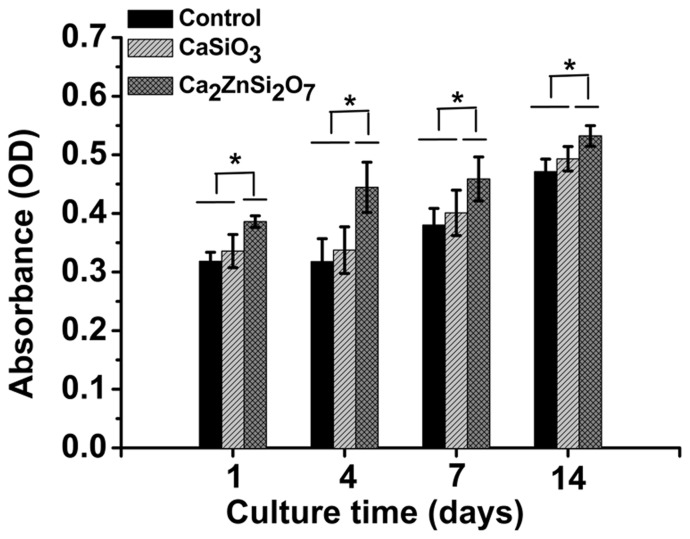
MC3T3-E1 cell proliferation on control, CaSiO_3_ coated and Ca_2_ZnSi_2_O_7_ coated substrates after 1, 4, 7 and 14 days in culture. Cell proliferation of MC3T3-E1 cells cultured on the different substrates was measured by MTT assay. Ca_2_ZnSi_2_O_7_ coating enhanced the proliferation of preosteoblasts. All experiments were repeated in triplicate *Ca_2_ZnSi_2_O_7_ coating compared with CaSiO_3_ coating or control, *p*<0.05. Data presented as mean ± SD (n = 6).

MC3T3-E1 cell proliferation on control, CaSiO_3_ and Ca_2_ZnSi_2_O_7_ coated substrates was determined using an MTT assay. A statistically significant increase in cell proliferation was measured on all samples with increasing culture time (Fig. 3). Cell proliferation was increased on days 1, 4, 7 and 14 on Ca_2_ZnSi_2_O_7_ coated substrate compared to either CaSiO_3_ coated substrate or control (*p*<0.05).

### Cell Differentiation and Mineralization

**Figure 4:**
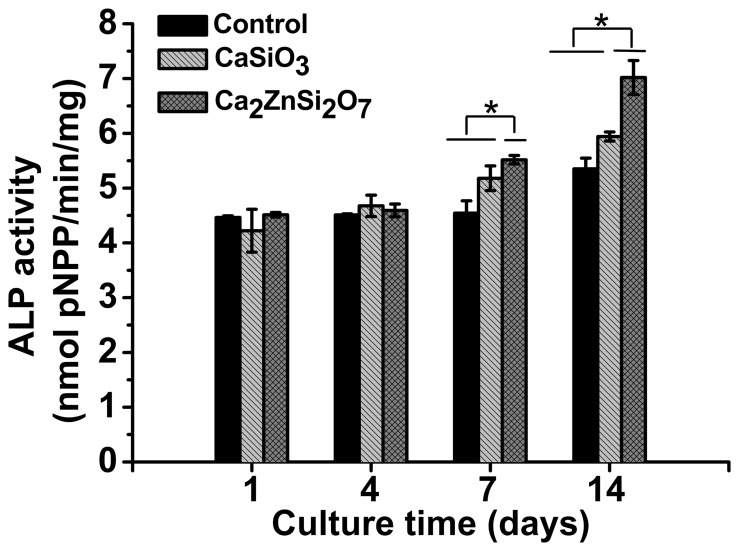
ALP activity in MC3T3-E1 cells cultured on control, CaSiO_3_ and Ca_2_ZnSi_2_O_7_ coated substratesfor 1, 4, 7 and 14 days. All experiments were repeated twice with n = 3 for each substrate. *Ca_2_ZnSi_2_O_7_ coating compared with CaSiO_3_ coating or control, *p*<0.05. Results are presented as mean ± SD.

Cell differentiation from pre-osteoblasts to osteoblasts revealed osteogenic functioning of MC3T3-E1 cells on all substrates. ALP assays were performed on MC3T3-E1 cells cultured on control, CaSiO_3_ and Ca_2_ZnSi_2_O_7_ coated substrates at various time points. ALP activity presented a similar profile for all samples, with increased activity throughout the culture duration (Fig. 4). For all conditions tested, there was no significant difference in ALP activity between days 1 and 4, after which levels rapidly increased up to day 14. ALP activity within cells incubated on Ca_2_ZnSi_2_O_7_ coating increased above those on the CaSiO_3_ coating and control (*p*<0.05 for CaSiO_3_ coating and control at day 7, 14). Results therefore indicate that Ca_2_ZnSi_2_O_7_ coating can promote cell maturation as reflected by increased ALP activity.

**Figure 5:**
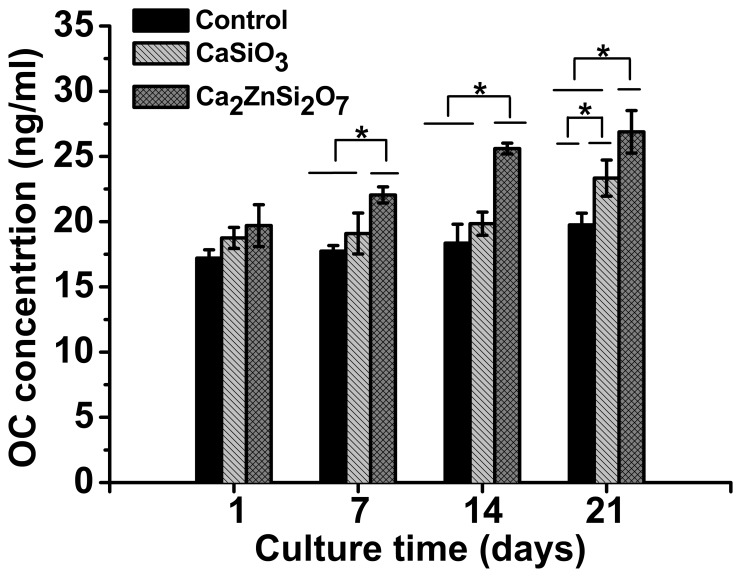
OC level in MC3T3-E1 cells cultured on control, CaSiO_3_ and Ca_2_ZnSi_2_O_7_ coated substrates for 1, 7, 14 and 21 days determined using ELISA assay. All experiments were repeated twice with n = 3 for each substrate.*Ca_2_ZnSi_2_O_7_ coating compared with CaSiO_3_ coating or control, *p*<0.05. Results presented are the mean ± SD.

Levels of the differentiation marker, OC, were investigated at days 1, 7, 14 and 21 of culture. As shown in Fig. 5, no significant difference in OC levels were found in MC3T3-E1 cells cultured on Ca_2_ZnSi_2_O_7_, CaSiO_3_ and control substrates at day 1. However, increases in OC were observed at days 7, 14 and 21 (*p*<0.05) in cells grown on the Ca_2_ZnSi_2_O_7_ coating compared to CaSiO_3_ coated substrates and control.

### Osteoblast-related Gene Expression

**Figure 6:**
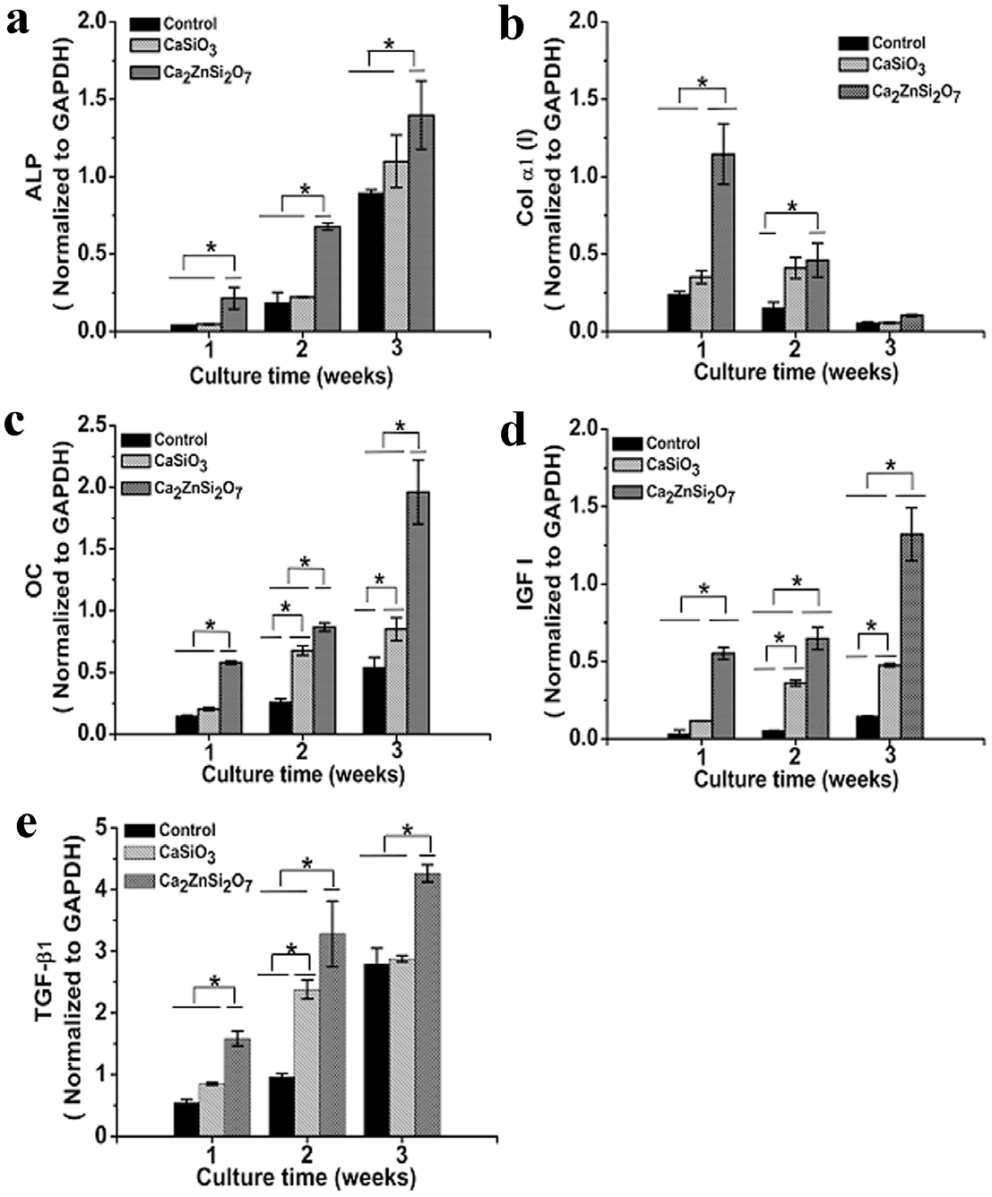
qRT-PCR analysis of the expression of different osteoblast MC3T3-E1 cell markers (a) ALP, (b) Col α1(I), (c) OC, (d) IGF-I and (e) TGF-β1 on control, CaSiO_3_ and Ca_2_ZnSi_2_O_7_ coated substrates at weeks 1, 2 and 3. *Ca_2_ZnSi_2_O_7_ coating compared with CaSiO_3_ coating and control,*p*<0.05. Results were normalized using GAPDH as a house keeping gene. Results presented are the mean ± SD (n = 3) of three independent experiments.

MC3T3-E1 cells were cultured for 1, 2 and 3 weeks on control, CaSiO_3_ and Ca_2_ZnSi_2_O_7_ coatings and expression of typical bone-related genes was examined using qRT-PCR (Fig. 6). ALP mRNA expression levels (Fig. 6a) in MC3T3 cells cultured on Ca_2_ZnSi_2_O_7_ coating steadily increased with time and were significantly higher (*p*<0.05) compared to cells cultured on CaSiO_3_ coating and control. These results are consistent with the previous ALP activity findings. procollagen α1(I) mRNA expression (Fig. 6b) decreased with time for all samples, but levels were significantly higher in cells on Ca_2_ZnSi_2_O_7_ coating in comparison to those on CaSiO_3_ coating and control (*p*<0.05 for CaSiO_3_ coating and control at week 1; *p*<0.05 for control alone at week 2). Osteocalcin mRNA expression (Fig. 6c) increased slightly over two weeks for all samples, after which levels rapidly increased up to week 3. In comparison to CaSiO_3_ coating and control, osteocalcin mRNA levels in cells grown on Ca_2_ZnSi_2_O_7_ coating were significantly increased (*p*<0.05 for CaSiO_3_ coating and control). IGF-I mRNA expression (Fig. 6d) increased with time for all samples although levels were significantly higher in cells grown on Ca_2_ZnSi_2_O_7_ coating compared to CaSiO_3_ coating and control (*p*<0.05 for CaSiO_3_ coating and control). TGF-β1 mRNA expression (Fig. 6e) increased with time for all samples and was significantly higher in cells grown on Ca_2_ZnSi_2_O_7_ coating compared to CaSiO_3_ coating and control (*p*<0.05 for CaSiO_3_ coating and control).

### *In vivo* Analysis

#### Histology

**Figure 7:**
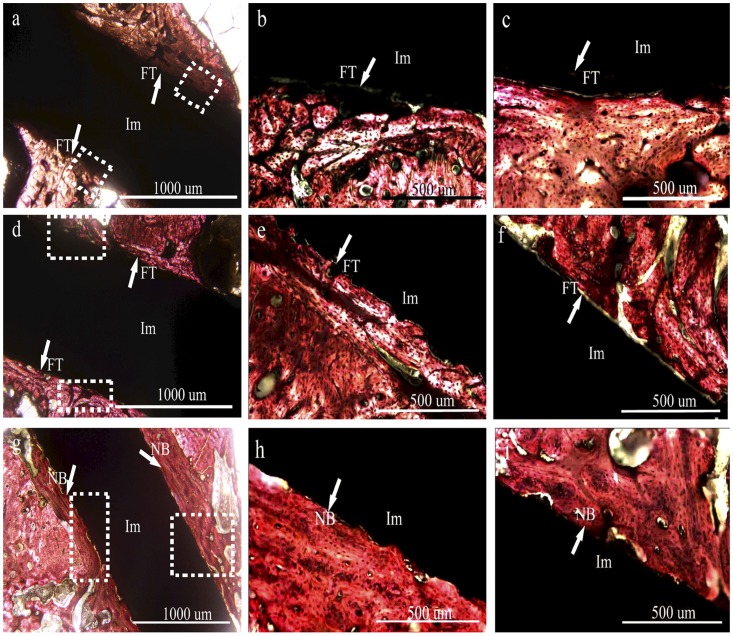
Histological morphology of the interface between implant and bone tissue after 1.5 month implantation in rabbit femurs. (a–c) control, (b) magnification of the left white square in panel (a), (c) magnification of the right white square in panel (a); (d–f) CaSiO_3_ coating, (e) magnification of the bottom white rectangle in panel (d), (f) magnification of the top white rectangle in panel (d), (g–i) Ca_2_ZnSi_2_O_7_ coating, (h) magnification of the left white rectangle in panel (g), (i) magnification of the right white square in panel (g). FT, fibrous tissue; Im, implant; NB, new bone.

The excellent *in vitro* properties of Ca_2_ZnSi_2_O_7_ coating led to analysis of its *in vivo* biocompatible properties following implantation into a rabbit femur defect model. No inflammation or other implant-associated complications were observed macroscopically or microscopically in any of the histopathology sections for the implanted Ca_2_ZnSi_2_O_7_ coatings throughout the experimental periods. After 1.5 month of implantation, new bone was primarily observed at the Ca_2_ZnSi_2_O_7_ coating periphery with direct contact to the implant surface in absence of a connective tissue layer (Fig. 7g–i). In contrast, fibrous tissue was clearly seen around the surface of the CaSiO_3_ coating (Fig. 7d–f) and control (Fig. 7a–c), resulting in markedly reduced bone-implant contact. New bone formation was sparse around these two implants.

#### Histomorphometry

**Figure 8:**
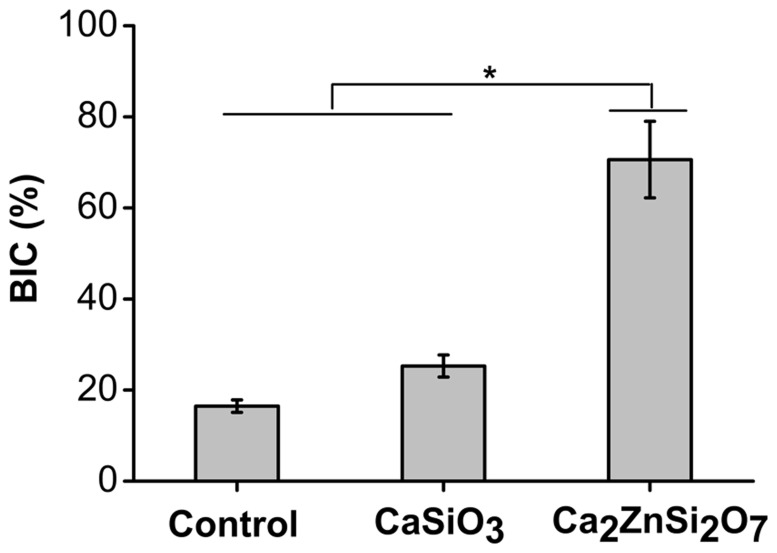
Bone-implant contact rate (BIC) of each group after 1.5 month insertion. It showed the mean BIC over the total implant length. For the Ca_2_ZnSi_2_O_7_ group, the BIC showed significant differences compared with CaSiO_3_ coating and control (*p<0.05). Results presented are the mean ± SD (n = 6) of two independent experiments.

The results of the histomorphometric measures are shown in Fig. 8. The bone-implant contact rate (BIC) was 70.61±8.42% in the Ca_2_ZnSi_2_O_7_ coating group, which was significantly (*p*<0.05) higher than the values of 25.29±2.43% observed in the CaSiO_3_ coating group and 16.47±1.39% in the control group.

## Discussion

It is well-known that biomaterial physicochemical characteristics are important in tissue engineering because of their direct effect on cellular response and ultimately tissue regeneration [23]. Surface modifications have been applied to metallic biomaterials in order to improve their wear properties, corrosion resistance, and biocompatibility [24], [25]. Our previous studies have shown the Zn-modified calcium silicate (Ca_2_ZnSi_2_O_7_) ceramic coating exhibits significantly improved stability in physiological solution and good bioactivity, as well as high bonding strength with the titanium alloy substrate [22]. We therefore conducted a more detailed study to examine and compare the *in vitro* and *in vivo* response of osteoblast-like MC3T3-E1 cells cultured on uncoated Ti-6Al-4V titanium (control), CaSiO_3_ coated and Ca_2_ZnSi_2_O_7_ coated substrates and investigate their capacity to conduct osteoregeneration after implantation into a rabbit femur defect model.

Biomaterial surface specifications can influence adsorption of biological molecules and, in the second stage, adsorption of cells [26]. Hence, adequate adhesion and spreading of cells are prerequisite for the interaction of cells with their substrate, which will determine subsequent cellular activities including proliferation and differentiation. Investigation into cell attachment found that MC3T3-E1 cells adhere to Ca_2_ZnSi_2_O_7_ coating with greater spread and number of filopodia projections anchored to the surface than those adhering to CaSiO_3_ coating or control, which indicates that Ca_2_ZnSi_2_O_7_ coating has a good bioactive surface favorable for cell adhesion and growth with good biocompatibility.

Ideally bioactive biomaterials need to interact actively with cells and stimulate cell growth [27]. MTT assay showed MC3T3-E1 cells could proliferate on all three samples. Furthermore, the proliferation ratio on Ca_2_ZnSi_2_O_7_ ceramic coating was higher than that on CaSiO_3_ coating or control at all time points, suggesting Ca_2_ZnSi_2_O_7_ coating modulates cell proliferation activity. Besides proliferation, the ability of preosteoblasts to differentiate on the biomaterials is an important stage that occurs before bone mineralization. ALP was used as a marker for early and mid-stages of osteoblast maturation and bone matrix production [28]–[30]. In our study, MC3T3-E1 cell ALP activity on Ca_2_ZnSi_2_O_7_ coating exhibited higher levels of expression than on CaSiO_3_ coating or control from day 7. This is in accordance with the results of ALP gene regulation, indicating Ca_2_ZnSi_2_O_7_ coating might possess a greater ability to stimulate preosteoblast differentiation compared to CaSiO_3_ coating and control. As expected, Zn ions appear to be the relevant inducers of ALP activity, since ALP is a Zn-dependent enzyme [31]. OC is secreted solely by osteoblasts and thought to play a role in cell mineralization and calcium ion homeostasis [32]. Our results show that Ca_2_ZnSi_2_O_7_ coating indeed markedly raises levels of OC over control levels at later periods in culture, which indicates their higher maturation state at this period. Further investigation into OC expression found mRNA levels followed a similar trend to OC levels, indicating that Zn-modified calcium silicate (Ca_2_ZnSi_2_O_7_) ceramic coating possesses a greater ability to promote osteoblast mineralization of MC3T3-E1 cells compared to CaSiO_3_ coating or control. Our findings are consistent with data reported by other research groups. Chesters [33] and Cousins [34] report that zinc is an essential trace element involved in diverse metabolic and cellular signaling pathways and is involved in the modulation of gene expression for proteins involved in bone formation. Ito *et al* also report that incorporation of zinc into calcium phosphate cement significantly promotes preosteoblast proliferation and differentiation *in vitro* [35]–[37]. More recently, Kwun *et al.* suggest zinc is involved in regulating the transcription of preosteoblast differentiation genes, such as Col-I, ALP, osteopontin and OC [38]. Hence, Zn is considered to be one promising agent for enhancing the bone-forming ability of implant materials, which can be achieved by controlling the release of Zn ions. Our studies suggest that Zn ions released form Ca_2_ZnSi_2_O_7_ coatings might play an important role in MC3T3-E1 cell proliferation and differentiation.

In the present study, MC3T3-E1 cells were used to investigate the influence of Ca_2_ZnSi_2_O_7_ coating on regulation of osteoblast-associated genes: ALP, Col α1(I) and OC by qRT-PCR. These genes are major phenotypic markers for preosteoblast differentiation during bone formation [39]. Col-I is the most abundant protein synthesized by active osteoblasts and is essential to mineral deposition, so its expression represents the start of osteoblast differentiation [40]. Col α1(I) mRNA expression (early marker, major extracellular collagenous protein for organic matrix formation) was upregulated in cells on Ca_2_ZnSi_2_O_7_ coating compared to CaSiO_3_ coating and control. The increase was pronounced during the early stage time points, which represent the beginning of osteoblast differentiation. Similar results were observed by Ehara *et al*, who found that calcium phosphate compounds cultured with MC3T3-E1 cells accelerated osteoblast differentiation in the early phase and promoted matrix production [41]. The results of this study demonstrate that at molecular level, osteoblast bone marker gene expression (ALP, Col α1(I), OC) on Ca_2_ZnSi_2_O_7_ coating is up-regulated, indicating Ca_2_ZnSi_2_O_7_ as a more favorable substrate for MC3T3-E1 cells with the potential for applications in bone tissue regeneration.

Growth factors have become an important component for tissue engineering and regenerative medicine [42]–[44]. In particular, IGF-I and TGF-β1 have great significance in bone tissue engineering [45]. IGF-I has been implicated as a regulator of osteoblast abundance to maintain bone matrix by enhancing osteoblast matrix production [46] and regulating osteoclast bone resorption [47]. TGF-β1 is considered another important regulator of osteoblast and osteoclast activity [48]. It is activated by resorbing osteoclasts which in turn attenuates further bone resorption by impairing osteoclastogenesis and promoting bone formation through chemotactic attraction of osteoblasts, enhancement of osteoblast proliferation and the early stages of differentiation with production of ECM proteins (including Col-I, osteopontin, OC) [49]. However, the effect of biomaterials on gene expressions of these factors in osteoblast cells has not been fully clarified. This study investigated mRNA expressions of IGF-I and TGF-β1 on the control, CaSiO_3_ and Ca_2_ZnSi_2_O_7_ coated substrates using qRT-PCR. We found that culture on Ca_2_ZnSi_2_O_7_ coating caused a significant increase in IGF-I and TGF-β1 mRNA expression in MC3T3-E1 cells, both of which are involved in the stimulation of bone formation and cell proliferation within osteoblast cells. Yamaguchi *et al.* also report that culture with zinc stimulates protein production of IGF-I, TGF-β1 or osteocalcin in osteoblastic MC3T3-E1 cells [50].

All the results obtained *in vitro* indicate that Ca_2_ZnSi_2_O_7_ coating is more favorable to the cellular activities of MC3T3-E1 cells, indicating that this coating might be suitable for bone regeneration and tissue engineering compared to CaSiO_3_ coating and control. The behavior of the bone tissue around Ca_2_ZnSi_2_O_7_ coating following implantation in rabbit femurs was also investigated. We found new bone was formed and in direct contact with the implants after 1.5 month of Ca_2_ZnSi_2_O_7_ coating implantation, in absence of fibrous tissue infiltration around the implant. In contrast, a wide band of fibrous tissue was clearly seen around the surface of the CaSiO_3_ coating and control. These observations were in line with the histomorphometrical data. Our results showed the direct bone contact with the implant (BIC) of Ca_2_ZnSi_2_O_7_ coating group was significantly greater than that for CaSiO_3_ coating and control after 1.5 month implantation. So, we concluded that Ca_2_ZnSi_2_O_7_ coatings have better bone integration properties after implantation, compared to CaSiO_3_ coating and control. This is consistent with the results obtained by Zreiqat *et al.*, who observed that the incorporation of Sr and Zn into their Ca–Si system induced *in vivo* osteoconductivity at 3 and 6 weeks following implantation in tibial bone defects in rats [51].

### Conclusions

Plasma-sprayed Ca_2_ZnSi_2_O_7_ coating enhanced MC3T3-E1 cell attachment, proliferation, differentiation and up-regulated bone marker gene expression of ALP, Col α1(I) and OC and growth factors genes of IGF-I and TGF-β1, compared to CaSiO_3_ coating and control. The *in vivo* study demonstrates further efficacy of Ca_2_ZnSi_2_O_7_ coating in modulating bone formation around the implant and enhancement of osseointegration in absence of fibrous tissue response. In conclusion, the biological properties observed for the investigated Ca_2_ZnSi_2_O_7_ coating strongly suggest it is a good candidate for orthopedic and dental implant fixation.
